# Mechanisms of Evolution in High-Consequence Drug Resistance Plasmids

**DOI:** 10.1128/mBio.01987-16

**Published:** 2016-12-06

**Authors:** Susu He, Michael Chandler, Alessandro M. Varani, Alison B. Hickman, John P. Dekker, Fred Dyda

**Affiliations:** a Laboratory of Molecular Biology, National Institute of Diabetes and Digestive and Kidney Diseases, National Institutes of Health, Bethesda, Maryland, USA; b Laboratoire de Microbiologie et Génétique Moléculaires, Centre National de la Recherche Scientifique, Toulouse, France; c Departamento de Tecnologia, Faculdade de Ciências Agrárias e Veterinárias de Jaboticabal, Universidade Estadual Paulista, Jaboticabal, São Paulo, Brazil; d Department of Laboratory Medicine, Clinical Center, Microbiology Service, National Institutes of Health, Bethesda, Maryland, USA

## Abstract

The dissemination of resistance among bacteria has been facilitated by the fact that resistance genes are usually located on a diverse and evolving set of transmissible plasmids. However, the mechanisms generating diversity and enabling adaptation within highly successful resistance plasmids have remained obscure, despite their profound clinical significance. To understand these mechanisms, we have performed a detailed analysis of the mobilome (the entire mobile genetic element content) of a set of previously sequenced carbapenemase-producing *Enterobacteriaceae* (CPE) from the National Institutes of Health Clinical Center. This analysis revealed that plasmid reorganizations occurring in the natural context of colonization of human hosts were overwhelmingly driven by genetic rearrangements carried out by replicative transposons working in concert with the process of homologous recombination. A more complete understanding of the molecular mechanisms and evolutionary forces driving rearrangements in resistance plasmids may lead to fundamentally new strategies to address the problem of antibiotic resistance.

## IMPORTANCE

The spread of antibiotic resistance among Gram-negative bacteria is a serious public health threat, as it can critically limit the types of drugs that can be used to treat infected patients. In particular, carbapenem-resistant members of the *Enterobacteriaceae* family are responsible for a significant and growing burden of morbidity and mortality. Here, we report on the mechanisms underlying the evolution of several plasmids carried by previously sequenced clinical *Enterobacteriaceae* isolates from the National Institutes of Health Clinical Center (NIH CC). Our ability to track genetic rearrangements that occurred within resistance plasmids was dependent on accurate annotation of the mobile genetic elements within the plasmids, which was greatly aided by access to long-read DNA sequencing data and knowledge of their mechanisms. Mobile genetic elements such as transposons and integrons have been strongly associated with the rapid spread of genes responsible for antibiotic resistance. Understanding the consequences of their actions allowed us to establish unambiguous evolutionary relationships between plasmids in the analysis set.

## INTRODUCTION

Carbapenem resistance among Gram-negative bacteria constitutes an urgent and serious threat to public health (1, 2, 3). In carbapenemase-producing *Enterobacteriaceae* (CPE) such as *Klebsiella pneumoniae*, the spread of resistance has been facilitated by the presence of a prevalent carbapenemase gene, *bla*_KPC_, within a transposon, Tn*4401*, on a transmissible plasmid (4, 5). This combination allows *bla*_KPC_ transfer not only between members of the *Enterobacteriaceae* family but also from one plasmid to another and even between plasmids and the bacterial chromosome. Following the 2011-2012 National Institutes of Health Clinical Center (NIH CC) outbreak of carbapenem-resistant *K. pneumoniae* (KPC^+^) (6), the NIH CC instituted a comprehensive surveillance program based on perirectal sampling of patients and targeted culture surveys of the hospital environment (7). The resulting collection of isolates provides a unique and valuable resource for understanding the mechanisms underlying the influx and transmission of antibiotic-resistant strains and the plasmids they carry within a hospital during a defined period. Whole-genome sequencing of these isolates using a combination of short- and long-read sequencing technologies has provided invaluable insight into several aspects of the hospital outbreak (6, 7, 8, 9).

Initially, the genetic data were used to generate a transmission map that accounted for the spread of the original KPC^+^ *K. pneumoniae* index strain to other patients during the outbreak, based on single nucleotide variations (SNVs) (6). Epidemiologic tracking following the outbreak using long-read PacBio single-molecule real-time (SMRT) sequencing had demonstrated that most of the subsequently identified KPC^+^ plasmids in patient isolates appeared to be unique and could not be linked easily to patient-to-patient transmission events (7). These PacBio plasmid reference assemblies also enabled high-accuracy transposon annotation (8). Analysis of transposon sequences demonstrated that among an important subset of plasmids, the most prevalent insertion sequence (IS) is IS*26*, and its mobility drives large-scale changes in plasmid structure. Unraveling the mechanisms behind these changes required analysis of the entire pool of genetic information from the sequenced hospital strains and revealed previously unrecognized genetic relationships between the plasmids involved (8).

More recent analysis has been performed on isolates collected longitudinally from two patients from the original 2011-2012 outbreak (patients 15 and 16), who have demonstrated persistent gastrointestinal colonization over the course of 2 to 4 years following the outbreak (9). High-quality PacBio reference assemblies of the plasmids carried by these isolates afforded a unique view of changes occurring in isolates over time in the natural context of colonization of a human host, and analysis revealed that substantial genetic rearrangements have occurred in the plasmids carried by these isolates (9). For patient 15, two KPC^+^ *K. pneumoniae* strains were isolated nearly 2 years apart, both of which carried three plasmids. Common to both strains was the pKpQIL plasmid from the original outbreak that carries the *bla*_KPC_ gene and confers carbapenem resistance. Genomic sequencing indicated that the two other plasmids in the 2013 strain were novel and composed entirely of rearranged DNA segments originating from the two additional plasmids of the patient’s original strain, KPNIH19 (9). For patient 16, longitudinal sampling over the course of 2011 to 2014 identified three different *bla*KPC^+^ isolates with seven distinct plasmid backbones (further details about the sampling protocol can be found in reference 9). Of six fully sequenced strains, all contained the pKpQIL plasmid or a related variant (9).

**FIG 1:**
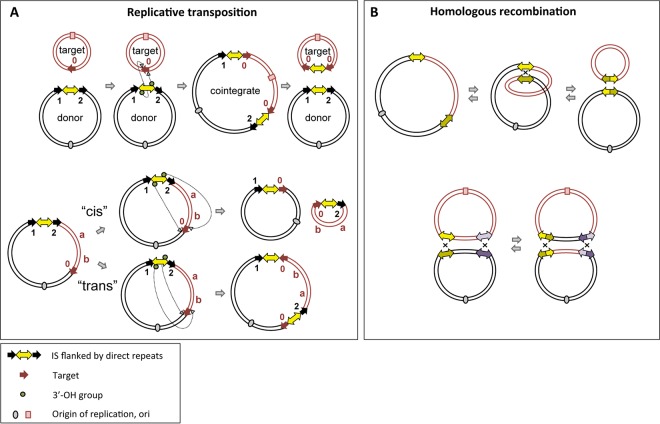
Schema of replicative transposition and homologous recombination. (A) Overview of replicative transposition. (Top) For intermolecular transposition, cleavage at both terminal inverted repeats (TIRs) of the insertion sequence (IS) (yellow double-headed arrow) results in nicks on both strands, generating 3′-OH groups (green circle) that attack the target site (red arrows). DNA replication generates a cointegrate containing a duplication of the IS and the target site; this can be subsequently resolved into a plasmid identical to the original donor plasmid and a modified target plasmid carrying an IS copy flanked by target site duplications (TSDs) arranged as direct repeats. (Bottom) For intramolecular transposition, the 3′-OH groups generated by cleavage at both TIRs can either attack the target site on the same strand (*cis*) or the opposite strand (*trans*). When in *cis*, DNA between the IS and target site becomes circularized and contains one IS copy and target site. In *trans*, DNA between IS and target site is instead inverted (“a b” becomes “b a”), bracketed by the original IS and a new copy in an inverted orientation. The target site is also duplicated but in inverted orientation, and each TSD is associated with one IS copy. Black arrows indicate potential TSDs from previous transposition events; different numbers represent different sequences. (B) Intramolecular/intermolecular homologous recombination (HR) (top) (forward and reverse arrows, respectively) and intermolecular exchange HR, resulting in DNA segment exchange (bottom). For intramolecular HR, a single crossover at two ISs generates two circular DNA products, each carrying one IS. For intermolecular exchange HR, both participating plasmids carry two ISs (yellow and purple double-headed arrows; identical copies in two plasmids are shown in the same color pattern but with different shades). Two crossovers at both identical ISs generate two recombined plasmids which have switched the DNA segment between the two ISs.

Here we have monitored the evolution of plasmids in two very different settings: isolates collected over several years from these two surviving patients colonized during the 2011-2012 NIH CC KPC^+^ outbreak (9) and a set of samples collected at the NIH CC from patients and from the hospital environment, again spanning several years (7). As a common hallmark of DNA transposition is the generation of target site duplications (TSDs) upon insertion into a new genomic location, as shown in Fig. 1A, TSDs can be used as tracers to track the movement of mobile elements and their length, orientation, and distribution (10) and can provide valuable information about transposition events. In combination with the known mechanism of homologous recombination (Fig. 1B), it is therefore possible to use the signatures of these processes to chart different pathways of plasmid evolution. Thus, we are able to propose the exact historical molecular events underlying plasmid rearrangements which provide a basis for understanding how antibiotic-resistant strains change over time, with significant implications for combating plasmid-mediated antimicrobial resistance.

## RESULTS

### Analysis of plasmids carried longitudinally in patients 15 and 16. (i) Patient 15.

**FIG 2:**
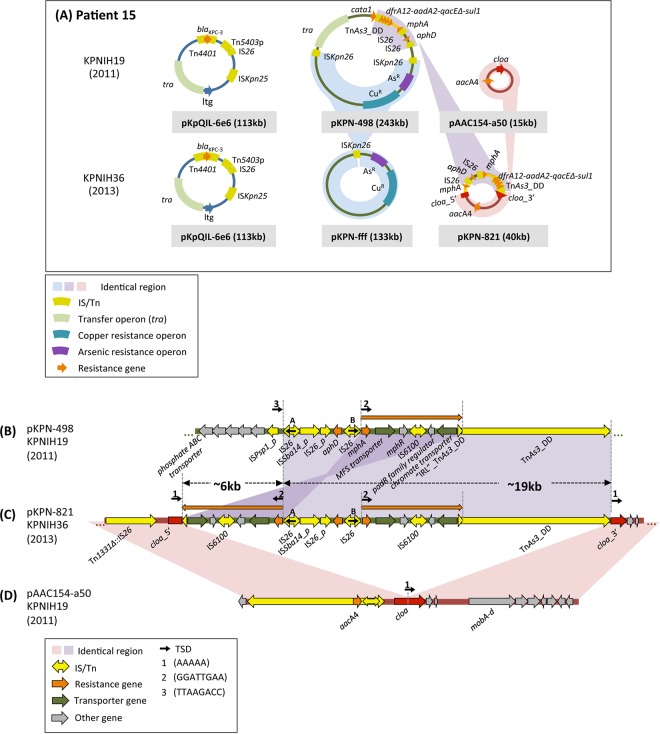
Overview and features of plasmids from patient 15. (A) Schematic of all the plasmids recovered from patient 15 isolates in 2011 and 2013 (6, 7, 9). The pKpQIL-6e6 plasmid was identified in 2011 and again in 2013 unchanged, and there were no SNVs. Homologous recombination between two IS*Kpn26* copies resulted in the downsizing of pKPN-498 (243 kb) to produce pKPN-fff (133 kb), and an insertion of 19 kb of DNA from pKPN-498 (with partial duplication) into pAAC154-a50 generated pKPN-821. (B to D) Alignment and details of plasmids from patient 15. Small black arrows topped by numbers represent target site duplications (TSDs) flanking various mobile elements. A ~25-kb rearranged segment from pKPN-498 (B) and the entire sequence of pAAC154-a50 (D) contribute to the generation of the hybrid plasmid pKPN-821 (C). Details of the proposed mechanism, involving Tn*As3*_DD and IS*26*, are shown in Fig. S2 in the supplemental material. The ~25-kb insertion disrupted the *cloa* gene while duplicating two transporter genes, one annotated as a chromate transporter and the other as an MFS (major facilitator superfamily) transporter.

We began by annotating the mobilome (the entire mobile genetic element content) present in the published genomic sequences of two KPC^+^ isolates cultured 2 years apart from patient 15 (9), both of which carry three plasmids (Fig. 2A; see Table S1 in the supplemental material). Full mobilome annotation allowed us to propose mechanisms that account for the transformation of the plasmids within the 2011 strain, KPNIH19, into two novel plasmids within the 2013 strain, KPNIH36.

*(a) pKPN-fff.* One of the new plasmids in the 2013 strain, pKPN-fff (133 kb), is a trimmed-down version of a substantially larger plasmid, pKPN-498 (243 kb), from the 2011 outbreak strain (Fig. 2A) (as already noted in reference 9). The DNA segment from pKPN-498 that has been lost is located between two directly repeated IS*Kpn26* copies (see Fig. S1 in the supplemental material), an arrangement that strongly suggests that pKPN-fff arose by intramolecular homologous recombination (Fig. 1B) between the two IS*Kpn26* mobile elements.

*(b) pKPN-821.* The second new plasmid of the 2013 isolate, pKPN-821 (Fig. 2A and C), is a hybrid composed of sequences originating from two plasmids of the outbreak parent strain (9). It consists of the entire sequence of outbreak strain plasmid pAAC154-a50 (Fig. 2D) interrupted in its *cloa* toxin gene by insertion of a 25-kb segment originating from pKPN-498 (Fig. 2B). Within this inserted segment, 19 kb is bounded by two mobile elements—an IS*26* copy (copy A in Fig. 2B and C) and a Tn*3* family transposon derivative related to Tn*As3* (designated Tn*As3*_DD [DD stands for deleted derivative] [see Fig. S2 in the supplemental material]). Although the Tn*As3*_DD sequence contains intact Tn*As3* transposase and resolvase genes, it does not possess terminal inverted repeats characteristic of transposons (Fig. S2). Instead, the transposon is bounded by 41-bp direct repeats, where the transposon left end (left inverted repeat [“IRL”]) is a direct repeat of the right end (right inverted repeat [IRR]) rather than an inverted repeat. Thus, Tn*As3*_DD is unlikely to be a canonically active mobile element. There is also an inverted duplication of 6 kb originating from within the 19-kb segment (marked with orange arrows in Fig. 2C). The 6-kb duplicated region is located between the left end of Tn*As3*_DD (“IRL”_Tn*As3*_DD) and a second IS*26* copy (copy B) in inverted orientation relative to copy A.

The rearrangements that led to the new plasmid, pKPN-821, are complex. However, several diagnostic sequence features suggest what processes may have occurred. The first revealing feature of pKPN-821 is that, at each junction of the pAAC154-a50-derived sequence and the 25-kb insert, there is a 5-bp DNA duplication (AAAAA; TSD 1 in Fig. 2C). Furthermore, at the junction between the insert and the 3′ part of the *cloa* gene (*cloa_*3′), the AAAAA repeat is directly abutted by Tn*As3*_DD. As replicative transposition of Tn*3* family members typically generates 5-bp direct duplications of the target site (ISfinder), a reasonable possibility is that the AAAAA repeat observed in pKPN-821 arose by intermolecular transposition by Tn*As3*_DD from pKPN-498 into pAAC154-a50 using an AAAAA sequence within the *cloa* gene as the target site. However, since the Tn*As3*_DD is not an intact transposon, this should not be a canonical transposition reaction. The simultaneous presence within pKPN-498 of Tn*As3*_DD encoding an active transposase and two IS*26* copies in inverted orientation suggests a pathway that might have led to the generation of pKPN-821. Specifically, as shown in Fig. S2B, transposition into the AAAAA target site of pAAC154-a50 by the IRL from one pKPN-498 plasmid copy and the IRR from a second pKPN-498 copy, followed by replication, would result in a fused plasmid structure that can be resolved by homologous recombination between two IS*26* copies to generate a closed plasmid identical to pKPN-821.

### (ii) Patient 16.

**FIG 3:**
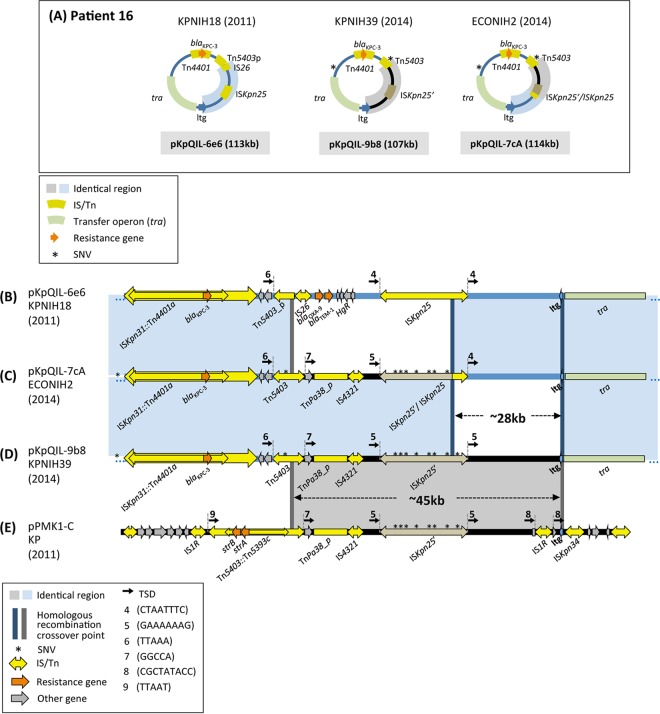
Overview and features of patient 16 plasmid rearrangements. (A) Schematic of the pKpQIL plasmids recovered from patient 16 isolates during the period from 2011 to 2014. SNVs are marked by asterisks. Three variants were recovered: pKpQIL-6e6 from the patient’s original strain (B), pKpQIL-7cA from *E. coli* strain ECONIH2 (C), and pKpQIL-9b8 from strain KPNIH39 (D). (E) Plasmid pPMK1-C contains a ~45-kb region that is also present in pKpQIL-9b8. In this plasmid, Tn*5403* has been disrupted within its *tnpA* gene by the insertion of Tn*5393c* that carries two resistance genes, *strA* and *strB*. In the gray ~45-kb region, pPMK1-C carries an IS*1R* copy upstream of the *ltg* gene that is flanked by 9-bp direct repeats (CGCTATACC; TSD 8), whereas pKpQIL-9b8 does not. Thus, it seems the recombination event with the pKpQIL plasmid occurred with a pPMK1-C ancestor prior to IS*1R* insertion.

Longitudinal perirectal sampling from patient 16 over the course of 4 years had identified—in addition to several other plasmids (see Table S1 in the supplemental material)—three variants of the pKpQIL plasmid (9), as shown schematically in Fig. 3A.

*(a) pKpQIL-9b8.* For one of the pKpQIL variants, pKpQIL-9b8 from *K. pneumoniae* KPNIH39, about half of the plasmid bears sequence highly similar to that of the original outbreak strain pKpQIL-6e6 (Fig. 3A), while the remaining 45-kb DNA segment originated elsewhere. Interestingly, as noted by Conlan et al. (9), the distinct DNA region in pKpQIL-9b8 (Fig. 3D, region highlighted in gray) has >99% similarity to a portion of pPMK1-C (Fig. 3E), a 70-kb plasmid from a *K. pneumoniae* strain isolated in a Nepali hospital during an sequence type 15 (ST15) *K. pneumoniae* outbreak in 2011 (11). At this time, we are aware of no other reported plasmids that contain this segment intact. In all three plasmids (pKpQIL-6e6 [Fig. 3B], pKpQIL-9b8 [Fig. 3D], and pPKM1-C [Fig. 3E]), there is a distinct region bracketed at its 5′ end by a Tn*5403* derivative and at the 3′ end by a lytic transglycosylase coding gene (designated *ltg*; Fig. 2 and 3). The Tn*5403* copy in pKpQIL-9b8 is intact, has an internal Tn*5393c* insertion in pPMK1-C, and has been truncated by an IS*26* copy in pKpQIL-6e6.

Thus, it seems likely that pKpQIL-9b8 of the 2014 *K. pneumoniae* isolate is the result of intermolecular homologous exchange between pKpQIL-6e6 and a plasmid related to pPMK1-C, where the two crossings took place at Tn*5403* and *ltg*. Although the Tn*5403* derivatives are disrupted in different ways in the recombining partners, there remains 344 bp of overlap (marked with dark gray bars in Fig. 3D and E) which should be sufficient for homologous recombination (12). Remarkably, one consequence of the proposed recombination event that led to pKpQIL-9b8 is the reconstitution of a full-length, and presumably once again active, Tn*5403* from its disrupted parts. Although Tn*5403* does not carry any antibiotic resistance genes (13), its regenerated activity returns it to the field of play as a potentially disruptive or reorganizing force via transposition within the 2014 pKpQIL plasmid variants.

*(b) pKpQIL-7cA.* The 2014 *Escherichia coli* pKpQIL variant, pKpQIL-7cA (Fig. 3C) from strain ECONIH2, is identical to pKpQIL-9b8 (Fig. 3D) except for a 28-kb DNA segment. This segment in pKpQIL-7cA is identical to a region from pKpQIL-6e6 of the initial 2011 outbreak strain. In all three pKpQIL plasmids (Fig. 3B to D), the 28-kb region is bracketed by an IS*Knp25* copy and the *ltg* gene. The most parsimonious explanation is that pKpQIL-7cA is the result of intermolecular exchange homologous recombination between pKpQIL-6e6 and pKpQIL-9b8, where one crossing is within IS*Kpn25* and the other is at *ltg* or its downstream region (since the sequence is identical in pKpQIL-6e6 and pKpQIL-9b8). However, we cannot rule out the possibility that pKpQIL-7cA is derived from homologous recombination between a pPMK1-C-like plasmid and pKpQIL-6e6.

The SNVs in the IS*Kpn25* copies (relative to pKpQIL-6e6, marked by asterisks in Fig. 3B to E) from the two recombining plasmids can be used to further refine the proposed recombination site. The SNVs of the 5′ part of the pKpQIL-7cA IS*Kpn25* copy are identical to those of the pKpQIL-9b8 copy, whereas the 3′ part is identical to the 2011 pKpQIL-6e6 copy. This therefore narrows the crossover site in IS*Kpn25* to a ~70-bp region within one of its passenger genes, *hsdM*, a DNA methylase that forms part of a restriction modification system (9).

### Evolutionary relationships of plasmids from CPE isolated in the NIH CC over a 3-year period.

Given the evidently dynamic nature of the plasmids isolated from these patients, we wondered whether there were other traceable sequence relationships among the 63 plasmids within the 20 previously isolated and fully sequenced KPC^+^ *Enterobacteriaceae* strains (see Table S2 in the supplemental material) that transcend the individual patient level and that might report on larger features of population structure, potentially even beyond the NIH Clinical Center where they were isolated (7). Our analysis of an earlier version of the plasmid pool established that several plasmids carried by different strains isolated from different patients at different time points are related to one another by plasmid rearrangements driven by IS*26* transposition (8). Although we have not been able to establish relationships between all of the 63 plasmids, we can distinguish two groups that clearly have related backbones.

### (i) pAAC154-related plasmid group.

**FIG 4:**
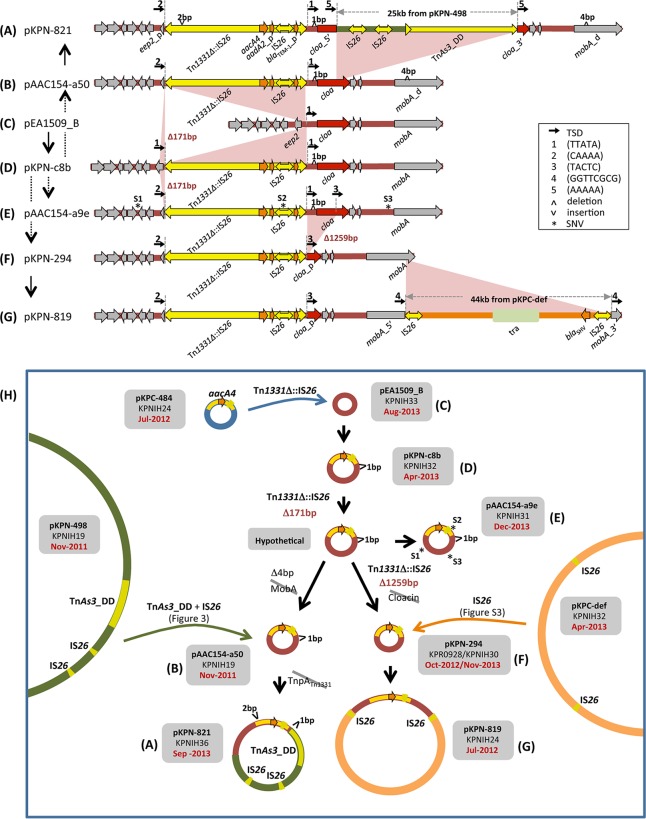
pAAC154 plasmid family within the NIH CC plasmid pool. (A to G) Schematics of the plasmid family members, showing their sequence relationships. Black vertical arrows mark the direction of evolution, where gray dashed lines indicate that there is an intermediate not detected within the plasmid pool. (A) Relative to the smallest family member, pEA1509_B (C), pKPN-821 has received a Tn*As3*_DD and IS*26*-mediated insertion of a ~25-kb fragment from pKPN-498 (Fig. 2) as well as an insertion of Tn*1331*Δ::IS*26*. There has also been a 2-bp DNA insertion within the *tnpA* gene of Tn*1331*Δ::IS*26* that presumably disables the transposon. (B) pAAC154-a50 was recovered from the original NIH outbreak strain. There has been a 4-bp deletion within the *mobA* gene (also seen in pKPN-821 [A]) (27). (C) pEA1509_B is identical to a previously reported *Enterobacter aerogenes* plasmid (GenBank accession no. FO203353.1). (D) pKPN-c8b is related to pEA1509_B (C) by the insertion of Tn*1331*Δ::IS*26*. Tn*1331*Δ::IS*26*, a Tn*3* family member, is a Tn*1331* derivative (28) in which an internal insertion of IS*26* partially deleted the *aadA2* and *bla*_TEM-1_ genes, and removed the truncated *tnpR* of Tn*1331* and the *bla*_OXA-9_ gene (8). Note that it has been previously referred to as IS*Swi1*-m2 (29). It carries an *aacA4* [*aac*(*6′*)*-Ib*] gene encoding an aminoglycoside 6′-*N*-acetyltransferase that confers resistance to several aminoglycosides such as amikacin and kanamycin (30). This gene is retained intact through all the subsequent steps of the evolutionary pathway. (E) Deletion of 171 bp at the left end of Tn*1331*Δ::IS*26* to form pAAC154-a9e is consistent with its intramolecular transposition. This reaction deleted one of the original flanking TTATA TSD sequences, labeled “1.” SNVs are numbered S1 to S3 and are marked by asterisks. (F) A second intramolecular transposition reaction by Tn*1331*Δ::IS*26* appears to have removed the second TTATA copy and truncated the *cloa* gene in pKPN-294. (G) Relative to pKPN-294 (F), pKPN-819 appears to have received an IS*26*-mediated insertion of 44 kb from pKPC-def (see Fig. S3 in the supplemental material). One of the genes within the 44-kb fragment, *bla*_SHV_, codes for a beta lactamase; the fragment also bears a conjugal transfer operon, possibly important for continued plasmid fitness as the *mobA* gene was disrupted when IS*26* transposed. (H) Summary of the transformation pathway of the plasmids from various patient strains. Note the temporal disconnect: pAAC154-a50 was isolated in November 2011 and pKPN-819 in July 2012. However, the ancestors of these plasmids were identified later.

The 2011 NIH outbreak strain KPNIH1 contained three plasmids, the smallest of which is pAAC154-a50 (and which is also found in strain KPNIH19 from patient 15 [Fig. 2A]). When we examined the entire sequenced plasmid pool, we found plasmids related to pAAC154-a50 ranging in size from 9 kb to 58 kb within eight patient isolates spanning the 2011-2013 collection period (Fig. 4A to G). Annotation and analysis of the mobile elements within these plasmid sequences revealed that the plasmids can be unambiguously placed into a branched evolutionary pathway that involves five transposition events and two small base pair insertions/deletions (Fig. 4H).

The smallest plasmid in the group is pEA1509_B (Fig. 4C), which is ~9 kb long. It was previously identified in an *Enterobacter aerogenes* clinical isolate (14) and differs only by 3 SNVs from *Citrobacter koseri* ATCC BAA-895 plasmid pCKO3, isolated in Maryland from a neonatal meningitis infection in 1983 indicating its long history (GenBank accession no. CP000823.1). This small plasmid then served as the target for insertion of a ~6-kb nested transposon, Tn*1331* containing a copy of IS*26* (designated Tn*1331*Δ::IS*26* [Fig. 4D]). The insertion occurred in the intergenic region between the cloacin (*cloa*) and entry exclusion protein 2 (designated *eep2*) coding genes, and resulted in a 5-bp TSD (TSD 1 in Fig. 4D) flanking Tn*1331*Δ::IS*26* in the new plasmid, pKPN-c8b.

Plasmid pKPN-c8b is related to two other plasmids within the pool by sequential deletion of two DNA regions. The deleted regions are directly abutted by Tn*1331*Δ::IS*26*, and thus, each transformation is consistent with an intramolecular transposition event carried out according to the “*cis*” pathway in Fig. 1A. First, Tn*1331*Δ::IS*26* transposition into the *eep2* gene led to the deletion of 171 bp of DNA flanking the transposon left end and removed the original 5-bp left TSD (Fig. 4D to E), generating plasmid pAAC154-a9e. This plasmid also contains three SNVs (shown as asterisks labeled S1 to S3) not present in any of the other plasmids in this phylogenetic group (the hypothetical SNV-less ancestor is shown in the middle of Fig. 4H). In the second intramolecular transposition reaction, Tn*1331*Δ::IS*26* deleted 1,259 bp of DNA flanking its right end (including the original right 5-bp TSD), thereby truncating the *cloa* gene (Fig. 4E to F) and generating pKPN-294.

The 14-kb product of the two transposon-mediated deletions, pKPN-294, was subsequently augmented by the insertion of a 44-kb fragment to generate pKPN-819 (Fig. 4F to G). The 44-kb block is bracketed by two IS*26* copies and is identical to a portion of another plasmid within the sequenced pool, pKPC-def from strain KPNIH32. The two IS*26* copies in the augmented plasmid are in turn flanked by 8-bp direct repeats (TSD 4 in Fig. 4G), collectively implicating IS*26* intermolecular transposition as the pathway for acquisition of the 44-kb fragment. Most plausibly, one of the three IS*26* copies within pKPC-def transposed into pKPN-294 initially to generate a cointegrate, followed by resolution by homologous recombination between two IS*26* copies that eliminated the remaining 72 kb of pKPC-def (see Fig. S3 in the supplemental material).

Two other plasmids within the pool appear to have branched from pKPN-c8b. Proceeding through the hypothetical ancestor of pAAC154-a9e without the three SNVs, a 4-bp deletion within the *mobA* gene generated pAAC154-a50 that was identified in the original outbreak strain (Fig. 4B). Plasmid pAAC154-a50 then received a 25-kb remodeled insertion from pKPN-498 as detailed in Fig. 2D, resulting in plasmid pKPN-821 (Fig. 4B to A).

### (ii) pKPC-like plasmid group.

**FIG 5:**
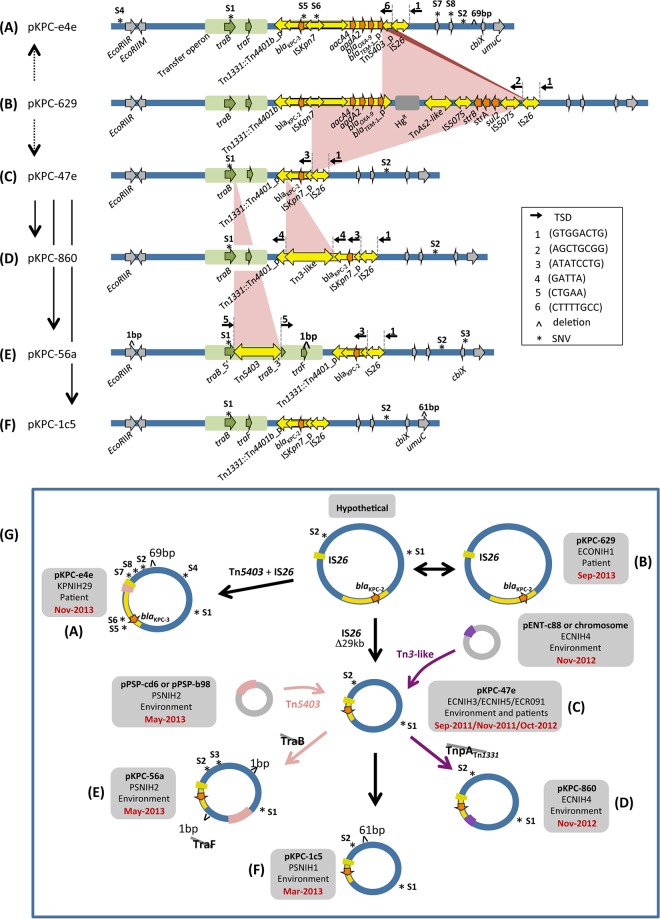
pKPC plasmid family within the NIH CC plasmid pool. (A to F) Schematics of the plasmid family members, showing their sequence relationships. Black vertical arrows mark the direction of evolution, where gray dashed lines indicate that there is an intermediate not detected within the plasmid pool. SNVs are numbered S1 to S8 and marked by asterisks. (A) Relative to pKPC-629 (B), pKPC-e4e is the product of IS*26*-mediated intramolecular transposition into Tn*1331*::Tn*4401b* and results in the deletion of the Tn*1331* right terminal repeat. pKPC-e4e has seven SNVs relative to pKPC-629 (B) and a 69-bp intergenic deletion not present in any of the other plasmids. Notably, pKPC-e4e has one SNV (S5) within the *bla*_KPC_ gene, which converts the *bla*_KPC-2_ allele to *bla*_KPC-3_. (B) pKPC-629 is the largest plasmid (~80 kb) within the family. (C) pKPC-47e has two diagnostic SNVs relative to the parent pKPC-629; these SNVs occur in *traB* (S1) and in a noncoding region (S2). Tn*4401* has been inactivated because IRR was destroyed. (D) Relative to pKPC-47e (C), pKPC-860 has received a Tn*3*-like transposon insertion. When this transposon sequence was subjected to a BLAST search against either the outbreak collection pool or the entire NCBI database, an identical copy without SNVs could be found only in other replicons from the same strain (ECNIH4). (E) Relative to pKPC-47e (C), pKPC-56a has received a Tn*5403* insertion; identical copies of this transposon were found only in other plasmids within strain PSNIH2. In addition to the SNVs observed in pKPC-47e, this plasmid has one more SNV in the *cbiX* gene (S3) and a single nucleotide deletion in each of two genes, a restriction endonuclease gene, *EcoRIIR*, and *traF*. (F) pKPC-1c5 has an additional 61-bp deletion in *umuC* compared to pKPC-47e (C). (G) Summary of the branched plasmid transformation pathway. The double-headed arrow relating pKPC-629 and the hypothetical plasmid indicates that it is impossible to establish the direction of the change. Note that pKPC-629 (September 2013) was identified later than the three instances of pKPC-47e (September and November 2011 and October 2012).

The original 2011 outbreak strain carried the *bla*_KPC_ gene within Tn*4401a* of a pKpQIL plasmid (7). However, there is a second plasmid group within the sequenced plasmid pool (Fig. 5) in which the *bla*_KPC_ gene is carried within Tn*4401b* transposons on pKPC-like plasmids. These pKPC-like plasmids have a broad host spectrum, which includes *E. coli*, *Enterobacter cloacae*, *Pantoea*, and *K. pneumoniae*.

The largest plasmid in the group, pKPC-629 from *E. coli* ECONIH1 (Fig. 5B), carries a copy of Tn*1331* in which its antibiotic resistance gene cluster that includes *bla*_OXA-9_ and *bla*_TEM-1_ has been augmented by the insertion of an active copy of Tn*4401b* bearing *bla*_KPC-2_ into the transposase *tnpA* gene of Tn*1331*. This plasmid does not carry two SNVs, S1 and S2, that are present in all other plasmids belonging to this group. Starting from a hypothetical derivative of pKPC-629 containing these two SNVs, it is possible to construct a branched evolution pathway (Fig. 5G) that accounts for the other five plasmids within the group.

In the first step on one branch, there appears to have been an IS*26*-mediated deletion of 29 kb of pKPC-629 to generate pKPC-47e (Fig. 5B to C); this product of intramolecular transposition in Tn*4401* occurs 472 bp upstream of the *bla*_KPC_ gene, leaving the resistance gene and its promoters (15) intact but rendering Tn*4401* inactive.

The pKPC-47e plasmid then underwent three separate transformations, two of which were transposon mediated. The resulting plasmids were all detected within environmental surveillance cultures rather than patients (7). Insertion of a Tn*3*-like transposon into the truncated *tnpA* gene of Tn*1331* gave rise to plasmid pKPC-860 from *E. cloacae* strain ECNIH4 (Fig. 5C to D) as evidenced by the generation of a 5-bp GATTA direct repeat on either side of the insertion (TSD 4). Five-base-pair direct repeats (TSD 5) are also observed flanking the site of a Tn*5403* insertion into the *traB* gene of pKPC-47e, a reaction that gave rise to pKPC-56a (Fig. 5G, parts C to E; *Pantoea* strain PSNIH2). The third transformation of pKPC-47e is a deletion of 61 bp in the *umuC* gene that generated pKPC-1c5 (Fig. 5C to F; *Pantoea* strain PSNIH1); this short deletion is not flanked by a mobile element, so it is not clear how it arose.

A separate branch of the evolutionary pathway appears to have generated plasmid pKPC-e4e from pKPC-629 (Fig. 5B to A). An IS*26*-mediated transposition reaction is most likely once again involved: pKPC-e4e has a truncated Tn*5403* immediately adjacent to the IS*26* copy that was involved in the pKPC-629-to-pKPC-47e transformation. The most straightforward explanation of these genetic features is that a Tn*5403* copy inserted into Tn*1331*::Tn*4401b* of pKPC-629, disrupting *bla*_TEM-1,_ prior to IS*26*-mediated deletion of 17 kb. The deleted region includes a mercury resistance operon as well as *strB*, *strA*, and *sul2* genes and most of the Tn*5403* copy.

## DISCUSSION

We have followed the evolution of plasmids in a set of previously sequenced CPE isolates from NIH Clinical Center patients (6, 7, 9). The availability of highly accurate plasmid assemblies for these strains based on long-read PacBio SMRT sequencing allows for the unambiguous and precise annotation of mobile elements. Importantly, as DNA transposition is generally not observed to be a chemically reversible reaction and often leaves detectable genomic rearrangements, tracking of successive events—in combination with information on homologous recombination events and SNVs—can establish the direction in time of the changes.

The analysis of plasmid sequences from two patients, patients 15 and 16, both colonized during the 2011-2012 NIH CC outbreak, has been particularly informative. In both cases, almost all of the plasmid changes could be interpreted in terms of replicative transposition events by a select set of mobile elements that appear to be particularly successful in CPE and of homologous recombination in which the copies of preexisting mobile elements served as crossover sequences. For patient 15, the plasmid sequences within the KPC^+^ strains isolated 2 years apart are self-contained: there has been no influx of new genes or DNA segments, and the plasmids in the 2013 isolate can be fully accounted for by replicative DNA transposition and homologous recombination reactions within the plasmids from 2011. However, the plasmid transformations do result in the loss of the conjugal transfer operon of one plasmid, and it would certainly be of interest to know whether the new plasmid pKPN-fff can be transferred in *trans* by the retained transfer operon present in pKpQIL-6e6. It would also be interesting to understand the consequences, if any, of the duplicated ~6-kb segment within the second new plasmid, pKPN-821, which provides extra copies of several genes including transporter and resistance genes. It is certainly possible that other rearrangements may have occurred but that the resulting plasmids were not selected or established, perhaps because of decreased fitness.

Additionally, point perirectal screening and culture methods may represent sparse sampling of the presumably complex underlying bacterial population, revealing only the most abundant descendants, and other reorganized plasmid structures may not have been sampled.

The variations observed in the pKpQIL plasmids from isolates from patient 16 were also straightforwardly interpretable, consisting entirely of homologous recombination events in which transposable elements provided the homology used in recombination. In contrast to the patient 15 plasmids, however, there has been the acquisition of an exogenous segment of DNA from another plasmid related to pPMK1-C from a 2011 *K. pneumoniae* outbreak in Nepal (9, 11).

Surprisingly, the recombination step leading to the 2014 plasmid pKpQIL-9b8 appeared to have reconstituted an intact wild-type Tn*5403* copy from two inactivated copies.

DNA transposition reactions dominate the evolution of the pAAC154 and pKPC group plasmids within the analyzed 2011-2014 NIH CC plasmid pool. For the former group, all the plasmid sequences are once again self-contained within the examined plasmid pool and can account for the observed transformations. pAAC154-like plasmids have been isolated in other hospitals around the world. For instance, plasmid pS15 carried by a carbapenem-resistant *K. pneumoniae* strain isolated in an Israeli hospital in 2006 is identical to pKPN-294 (Fig. 4F) except for an insertion of Tn*4401a* into the *mobA* gene with characteristic 5-bp direct repeats (16).

**FIG 6:**
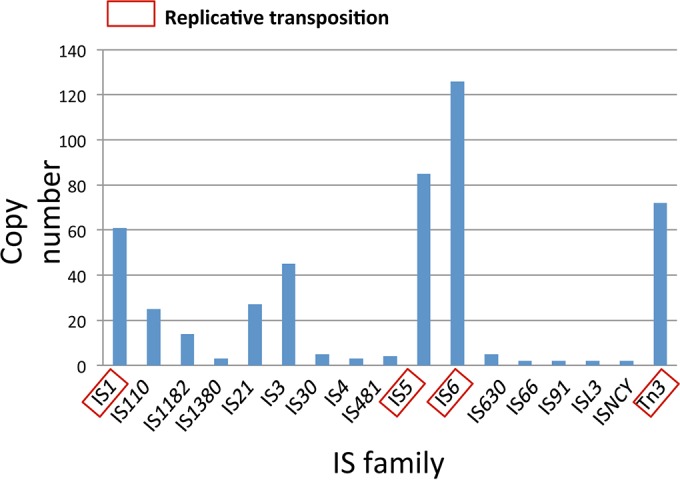
Classification of insertion sequences and transposons within the plasmid pool from 20 KPC^+^ *Enterobacteriaceae* strains isolated in the NIH Clinical Center from 2011 to 2013. The boxed IS families are those known (8, 17, 31, 32) to transpose using a replicative transposition mechanism. The complete list of strains analyzed can be found in Table S2 in the supplemental material.

The evolution of the second pKPC group involves the intramolecular transposition of IS*26* and intermolecular transposition by two mobile elements, Tn*5403* and a Tn*3*-like transposon, which are also resident on other plasmids within the clinical isolate collection. These transposons belong to the Tn*3* family, which is known to transpose by replicative transposition (17, 18), and detailed annotation of transposable elements in the entire plasmid collection revealed that replicative transposons represented a dominant component (Fig. 6).

The plasmids identified at different time points in patients 15 and 16 appear to represent consecutive steps in plasmid evolution, as might be expected. We were somewhat surprised to find that when the plasmids among our two analyzed groups were examined—isolated from different patients and the hospital environment—there was little correlation between the date of isolate collection and location on the inferred evolutionary tree (Fig. 4H and 5G). As we believe we have unambiguously established the sequence of the plasmid transformation events, a reasonable explanation for such temporal incongruities is that the ancestors continue to coexist alongside the progeny resulting from the identified successive transpositions within the larger population structure. It should be emphasized that though our method establishes the sequence of events, it does not establish the location or timing of events, and we do not suggest that our analysis can establish whether the identified transformations took place within the NIH Clinical Center; indeed this interpretation would be contradicted by epidemiologic evidence for many of the isolates (6, 7, 9). Rather, it is highly likely that our analysis represents a view into a globally structured plasmid population, as sampled in one hospital.

A natural question raised by our analysis is what are the evolutionary forces driving the plasmid transformations we have characterized. Are these random, selectively neutral changes—snapshots of which have been captured at the time of sampling—or are there specific features of the fitness landscape that lead to the prevalence of certain rearrangements rather than others? It would clearly be very interesting to determine what effects the changing genomic context of certain genes might have on gene expression, strain fitness, or antibiotic resistance. Potentially relevant genes include *bla*_KPC_ itself within the pKPC plasmid group, and the transporters within the 6-kb region that are duplicated in the transformation of pKPN-498 to pKPN-821 in patient 15.

It appears that two particular types of mobile elements, IS*26* and members of the Tn*3* transposon family, have played dominant roles in the evolution of the KPC^+^ strains we studied, for reasons that are not yet clear. A reported property of Tn*3* (19) is transposition immunity that should confer some protection against further transposition by Tn*3* into a replicon that already has an integrated copy. Immunity, a statistical and distance-dependent property, has been studied in detail for Tn*7* (20, 21) and bacteriophage Mu (22, 23) and has been shown to be dependent on a transposon-encoded ATPase. However, there is no evidence that Tn*3* encodes an ATPase (19), and how Tn*3* might confer immunity is not understood. Within the isolate collection examined here, there are instances of multiple Tn*3*-like copies in the same replicon; however, with one exception, these correspond to different members of the Tn*3* family. It would be interesting to determine experimentally whether Tn*3*-like elements display any measurable transposition immunity in KPC^+^ strains and, if they do, what is the mechanistic basis of such property and its limitations.

In contrast to Tn*3* transposons, IS*26* has no known transposition-suppressing properties. On the contrary, it has been reported that a replicon already containing an IS*26* copy is a favored target for further IS*26* integration (24). The mechanism at work is again unknown, but the unusually large number of IS*26* copies in certain replicons suggests that it may indeed occur in nature.

The rapidly decreasing cost of high-quality, long-read sequencing will enable the type of analysis described here to be applied more broadly to the problem of how resistance plasmids evolve in patients, hospitals, and the environment. Such knowledge will, in turn, facilitate better understanding of the underlying fitness landscapes driving the observed plasmid rearrangements and perhaps lead to new ways of addressing the problem of multiantibiotic resistance.

## MATERIALS AND METHODS

Plasmid sequences of longitudinally carried strains in patients 15 and 16 as well as the sequences of the 20 NIH CC isolates in this paper are from the NCBI public database (lists shown in Tables S1 and S2 in the supplemental material). Full annotation of the plasmid mobilome (the entire mobile genetic element content) was carried out using the ISfinder reference database (the curated database of prokaryotic insertion sequences [IS]) (25) and its associated annotation software, ISsaga (http://issaga.biotoul.fr/ISsaga2/issaga_index.php) (26). SnapGene 3.1.2 was used to visualize the annotation results.

## SUPPLEMENTAL MATERIAL

Figure S1  Plasmid alignment of pKPN-498 and pKPN-fff. Identical sequences are highlighted in blue. One notable consequence of homologous recombination between two IS*Kpn26* copies in pKPN-498 to generate pKPN-fff is the loss of the genes of the conjugative transfer operon. Other genes that have been lost include operons corresponding to a phosphate ABC transporter, a peptide ABC transporter, an iron transporter, and a klebicin B bacteriocidin gene cluster. Download Figure S1, TIF file, 0.03 MB

Figure S2  Organization of Tn*As3*_DD and proposed mechanism of formation of pKPN-821 from Tn*As3*_DD and two copies of pKPN-498. (A) The reference copy of Tn*As3* in the ISfinder database has 19 passenger genes. Here, the copy in pKPN-498 is truncated at its left end but retains the right part of the transposon carrying the resolvase and transposase genes and IRR. The 41-bp left end is probably the remaining transposon end of another decayed Tn*3*. The bases in IRL that differ from those in IRR are shown in green in Tn*As3* and in red in Tn*As3*_DD. (B) Transposition into the AAAAA target site of pAAC154-a50 by the IRL from one pKPN-498 plasmid copy and the IRR from a second pKPN-498 copy. Following the canonical replicative transposition processes of nicking (B), strand transfer (C), and replication (D), the target plasmid would expand in size by amplifying the DNA taking 3′-OH groups attached to the DNA strand in target plasmid as primers and using the donor plasmids as templates. According to DNA polarity, bidirectional replication (shown with black arrows) would fill in AAAAA pentanucleotides, IRL_Tn*As3*_DD, 6-kb inverted-duplication region (orange), IS*26_*A, 19-kb DNA region, IS*26*_B, and so on from the left 3′-OH group, as well as TTTTT pentanucleotides, Tn*As3*_DD, 6-kb inverted-duplication region (orange), IS*26*_A and so on from the right 3′-OH group. Homologous recombination between two IS*26* copies (A and B) would generate the closed plasmid pKPN-821 (E). An alternative possibility (not shown) is that transposition occurs between only two plasmids where the two pKPN-498 copies exist as a single plasmid dimer. Download Figure S2, TIF file, 0.1 MB

Figure S3  One possible mechanism of formation of pKPN-819 from pKPC-def and pKPN-294. One of the three IS*26* copies (shown as IS*26*_1) within pKPC-def may transpose into the GGTTCGCG octanucleotide (octanucleotide labeled 4) in pKPN-294 to initially generate a cointegrate with the duplication of IS*26* (duplicated copy is designated IS*26*_1′) and the octanucleotide labeled 4. Homologous recombination between IS*26*_1 and IS26_2 would eliminate the remaining 72 kb of pKPC-def while maintaining the 44-kb DNA segment. Notably, this segment carries a conjugal transfer operon and *bla*_SHV_. Download Figure S3, TIF file, 0.1 MB

Table S1  List of plasmids from longitudinally carried strains in patients 15 and 16 from 2011 to 2014. The plasmids analyzed in this study are labeled with an asterisk. Table S1, DOCX file, 0.02 MB

Table S2  Complete list of plasmids from 20 KPC^+^ *Enterobacteriaceae* strains isolated in the NIH Clinical Center from 2011 to 2013. The plasmids analyzed in this study are labeled with an asterisk. Table S2, DOCX file, 0.03 MB
