# Supplementary figures and images for: Mechanisms of Evolution in High-Consequence Drug Resistance Plasmids
Source: mBio. 2016 Dec 6;7(6):e01987-16. doi: 10.1128/mBio.01987-16 (PMC5142620; doi:10.1128/mBio.01987-16)

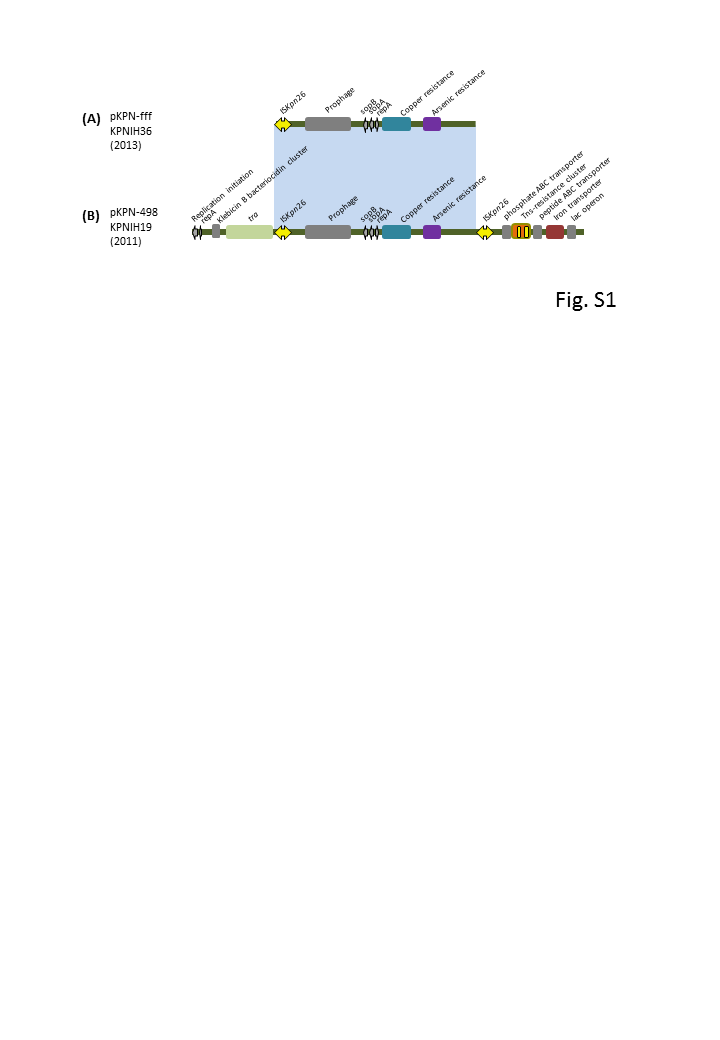

Supplement: Figure S1 — Plasmid alignment of pKPN-498 and pKPN-fff. Identical sequences are highlighted in blue. One notable consequence of homologous recombination between two ISKpn26 copies in pKPN-498 to generate pKPN-fff is the loss of the genes of the conjugative transfer operon. Other genes that have been lost include operons corresponding to a phosphate ABC transporter, a peptide ABC transporter, an iron transporter, and a klebicin B bacteriocidin gene cluster. Download [file mbo006163088sf1.tif]

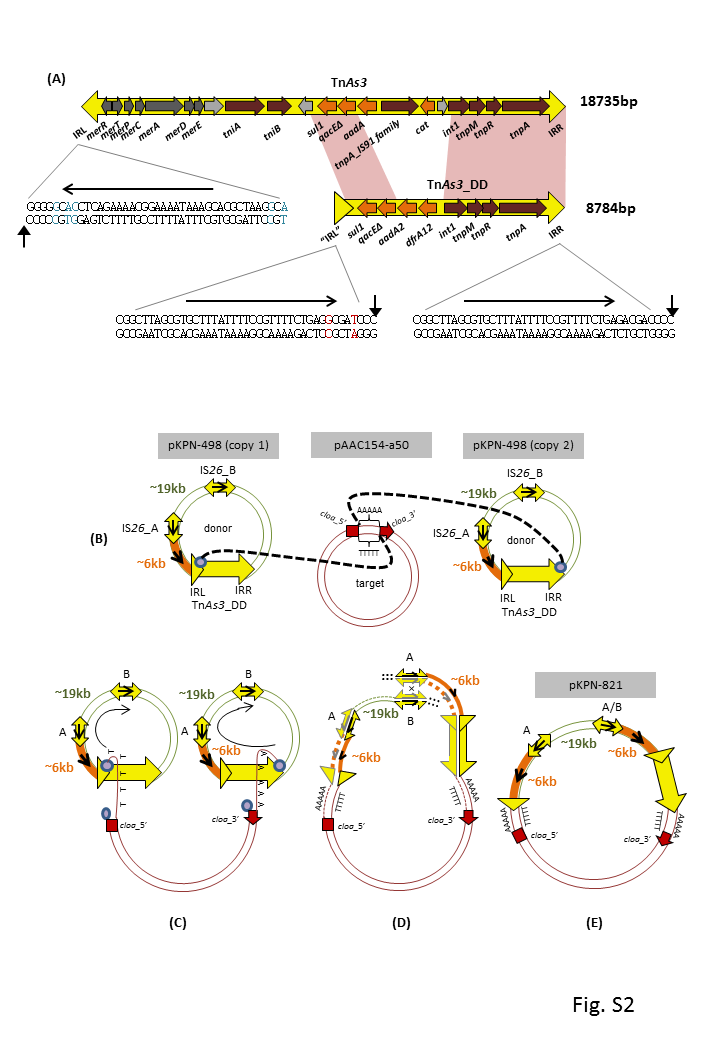

Supplement: Figure S2 — Organization of TnAs3_DD and proposed mechanism of formation of pKPN-821 from TnAs3_DD and two copies of pKPN-498. (A) The reference copy of TnAs3 in the ISfinder database has 19 passenger genes. Here, the copy in pKPN-498 is truncated at its left end but retains the right part of the transposon carrying the resolvase and transposase genes and IRR. The 41-bp left end is probably the remaining transposon end of another decayed Tn3. The bases in IRL that differ from those in IRR are shown in green in TnAs3 and in red in TnAs3_DD. (B) Transposition into the AAAAA target site of pAAC154-a50 by the IRL from one pKPN-498 plasmid copy and the IRR from a second pKPN-498 copy. Following the canonical replicative transposition processes of nicking (B), strand transfer (C), and replication (D), the target plasmid would expand in size by amplifying the DNA taking 3′-OH groups attached to the DNA strand in target plasmid as primers and using the donor plasmids as templates. According to DNA polarity, bidirectional replication (shown with black arrows) would fill in AAAAA pentanucleotides, IRL_TnAs3_DD, 6-kb inverted-duplication region (orange), IS26_A, 19-kb DNA region, IS26_B, and so on from the left 3′-OH group, as well as TTTTT pentanucleotides, TnAs3_DD, 6-kb inverted-duplication region (orange), IS26_A and so on from the right 3′-OH group. Homologous recombination between two IS26 copies (A and B) would generate the closed plasmid pKPN-821 (E). An alternative possibility (not shown) is that transposition occurs between only two plasmids where the two pKPN-498 copies exist as a single plasmid dimer. Download [file mbo006163088sf2.tif]

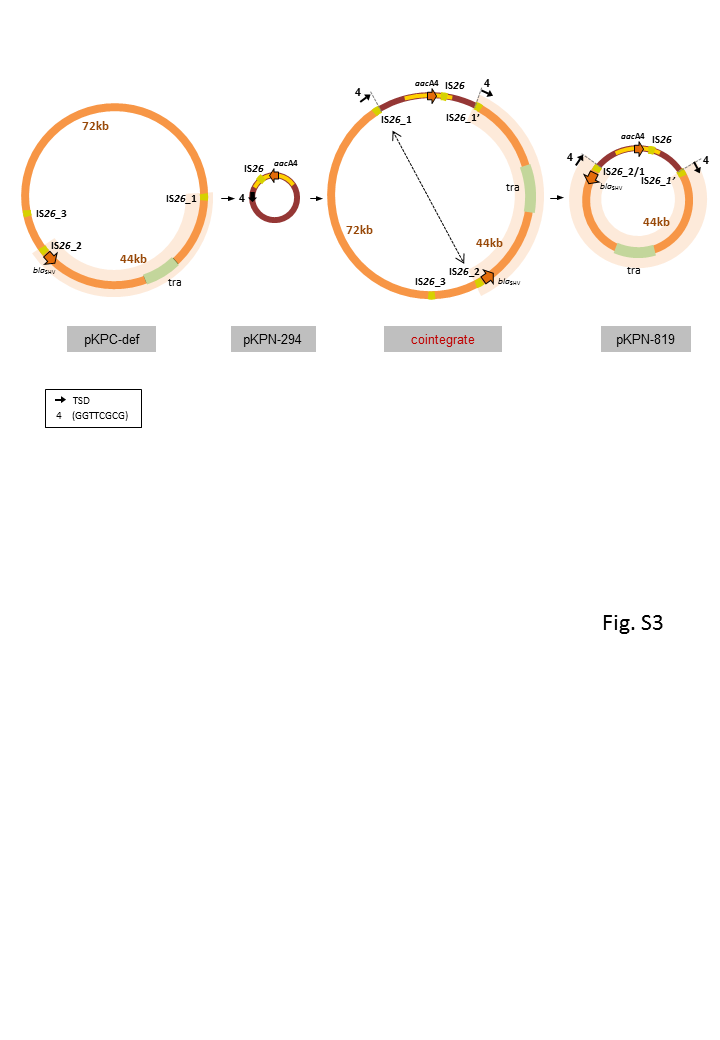

Supplement: Figure S3 — One possible mechanism of formation of pKPN-819 from pKPC-def and pKPN-294. One of the three IS26 copies (shown as IS26_1) within pKPC-def may transpose into the GGTTCGCG octanucleotide (octanucleotide labeled 4) in pKPN-294 to initially generate a cointegrate with the duplication of IS26 (duplicated copy is designated IS26_1′) and the octanucleotide labeled 4. Homologous recombination between IS26_1 and IS26_2 would eliminate the remaining 72 kb of pKPC-def while maintaining the 44-kb DNA segment. Notably, this segment carries a conjugal transfer operon and blaSHV. Download [file mbo006163088sf3.tif]
