# Supplementary material for: Mechanisms of Evolution in High-Consequence Drug Resistance Plasmids
Source: mBio. 2016 Dec 6;7(6):e01987-16. doi: 10.1128/mBio.01987-16 (PMC5142620; doi:10.1128/mBio.01987-16)
Supplement: Table S1 — List of plasmids from longitudinally carried strains in patients 15 and 16 from 2011 to 2014. The plasmids analyzed in this study are labeled with an asterisk. [file mbo006163088st1.docx]

| **Patient no.** | **Year of isolation** | **Genus and species** | **MLST** | **Strain** | **Plasmid** | **Genbank access no.** |
| --- | --- | --- | --- | --- | --- | --- |
| 15 | 2011 | *Klebsiella pneumoniae* | ST258 | KPNIH19 | *pAAC154-a50 | AKAJ00000000 |
|  |  |  |  |  | *pKPN-498 |  |
|  |  |  |  |  | *pKpQIL-6e6 |  |
|  | 2013 | *Klebsiella pneumoniae* | ST258 | KPNIH36 | *pKPN-821 | CP014648 |
|  |  |  |  |  | *pKPN-fff | CP014649 |
|  |  |  |  |  | *pKpQIL-6e6 | CP014650 |
| 16 | 2011 | *Klebsiella pneumoniae* | ST258 | KPNIH18 | pAAC154-a50 | AKAI00000000 |
|  |  |  |  |  | pKPN-498 |  |
|  |  |  |  |  | *pKpQIL-6e6 |  |
|  | 2014 | *Klebsiella pneumoniae* | ST37 | KPNIH39 | *pKpQIL-9b8 | CP014765 |
|  |  |  |  |  | pKPN-704 | CP014764 |
|  |  |  |  |  | pKPN-332 | CP014763 |
|  | 2014 | *Escherichia coli* | ST127 | ECONIH2 | *pKpQIL-7cA | CP014669 |
|  |  |  |  |  | pECO-bc6 | CP014668 |

Table S1: List of plasmids from longitudinally carried strains in patient 15 and 16 from 2011 to 2014. Those analyzed in this study are labeled “*”.
