# Supplementary material for: Mechanisms of Evolution in High-Consequence Drug Resistance Plasmids
Source: mBio. 2016 Dec 6;7(6):e01987-16. doi: 10.1128/mBio.01987-16 (PMC5142620; doi:10.1128/mBio.01987-16)
Supplement: Table S2 — Complete list of plasmids from 20 KPC+ Enterobacteriaceae strains isolated in the NIH Clinical Center from 2011 to 2013. The plasmids analyzed in this study are labeled with an asterisk. [file mbo006163088st2.docx]

| **Patient no.** | **Date of isolation** | ***Genus and species*** | **Strain** | **Plasmid** | **Genbank access no.** |
| --- | --- | --- | --- | --- | --- |
| 1 | Jun-11 | *Klebsiella pneumoniae* | KPNIH1 | *pAAC154-a50 | CP008828 |
|  |  |  |  | *pKPN-498 | CP008829 |
|  |  |  |  | *pKpQIL-6e6 | CP008830 |
| 5 | Aug-11 | *Klebsiella pneumoniae* | KPNIH10 | *pAAC154-a50 | CP007728 |
|  |  |  |  | *pKPN-498 | CP007729 |
|  |  |  |  | *pKpQIL-6e6 | CP007730 |
| 5 | Sep-11 | *Enterobacter cloacae* | ECNIH3 | pENT-576 | CP008898 |
|  |  |  |  | pENT-8a4 | CP008899 |
|  |  |  |  | pENT-d4a | CP008900 |
| Env | Nov-11 | *Enterobacter cloacae* | ECNIH5 | pENT-22e | CP009855 |
|  |  |  |  | pENT-784 | CP009856 |
|  |  |  |  | pENT-d0d | CP009857 |
|  |  |  |  | *pKPC-47e | CP009858 |
| A | Jan-12 | *Klebsiella pneumoniae* | KPNIH27 | pKEC-dc3 | CP007732 |
|  |  |  |  | pKPN-068 | CP007733 |
|  |  |  |  | pKPN-262 | CP007734 |
|  |  |  |  | pKPN-a41 | CP007735 |
|  |  |  |  | pKPN-b0b | CP007736 |
| Env | Jan-12 | *Citrobacter freundii* | CFNIH1 | pKEC-a3c | CP007558 |
| Env | Jan-12 | *Enterobacter cloacae* | ECNIH2 | pKEC-39c | CP008824 |
|  |  |  |  | pKPC-272 | CP008825 |
|  |  |  |  | pKPC-f91 | CP008826 |
| B | Jul-12 | *Klebsiella pneumoniae* | KPNIH24 | pKPC-484 | CP008798 |
|  |  |  |  | *pKPN-819 | CP008799 |
|  |  |  |  | pKPN-e44 | CP008800 |
| D | Oct-12 | *Enterobacter cloacae* | ECR091 | pENT-08e | CP008906 |
|  |  |  |  | pENT-4bd | CP008907 |
|  |  |  |  | *pKPC-47e | CP008908 |
| C | Oct-12 | *Klebsiella oxytoca* | KONIH1 | pKOX-137 | CP008789 |
|  |  |  |  | pKOX-86d | CP008790 |
|  |  |  |  | pKPC-727 | CP008791 |
| E | Oct-12 | *Klebsiella pneumoniae* | KPR0928 | *pKPN-294 | CP008832 |
|  |  |  |  | pKpQIL-531 | CP008833 |
| Env | Nov-12 | *Enterobacter cloacae* | ECNIH4 | pENT-c88 | CP009851 |
|  |  |  |  | pENT-e56 | CP009852 |
|  |  |  |  | *pKPC-860 | CP009853 |
| Env | Mar-13 | *Pantoea* | PSNIH1 | *pKPC-1c5 | CP009881 |
|  |  |  |  | pPSP-26e | CP009882 |
|  |  |  |  | pPSP-a3e | CP009883 |
|  |  |  |  | pPSP-ee2 | CP009884 |
| **Patient no.** | **Date of isolation** | ***Genus and species*** | **Strain** | **Plasmid** | **Genbank access no.** |
| F | Apr-13 | *Klebsiella pneumoniae* | KPNIH32 | *pKPC-def | CP009776 |
|  |  |  |  | pKPN-a68 | CP009777 |
|  |  |  |  | *pKPN-c8b | CP009778 |
| Env | May-13 | *Pantoea* | PSNIH2 | *pKPC-56a | CP009867 |
|  |  |  |  | pPSP-100 | CP009868 |
|  |  |  |  | pPSP-75c | CP009869 |
|  |  |  |  | pPSP-b98 | CP009870 |
|  |  |  |  | pPSP-cd6 | CP009871 |
| G | Aug-13 | *Klebsiella pneumoniae* | KPNIH33 | *pEA1509_B | CP009772 |
|  |  |  |  | pKPC-63d | CP009773 |
|  |  |  |  | pNJST258N3-62b | CP009774 |
| H | Sep-13 | *Escherichia coli* | ECONIH1 | pECO-824 | CP009860 |
|  |  |  |  | pECO-b75 | CP009861 |
|  |  |  |  | *pKPC-629 | CP009862 |
| I | Nov-13 | *Klebsiella pneumoniae* | KPNIH30 | *pKPN-294 | CP009873 |
|  |  |  |  | pKPN-b9c | CP009874 |
|  |  |  |  | pKpQIL-531 | CP009875 |
| J | Nov-13 | *Klebsiella pneumoniae* | KPNIH29 | *pKPC-e4e | CP009864 |
|  |  |  |  | pKPN-80a | CP009865 |
| K | Dec-13 | *Klebsiella pneumoniae* | KPNIH31 | *pAAC154-a9e | CP009877 |
|  |  |  |  | pKPN-852 | CP009878 |
|  |  |  |  | pKPN-c22 | CP009879 |

Table S2. Complete list of plasmids from 20 KPC^+^ Enterobacteriaceae strains isolated in the NIH Clinical Center from 2011 to 2013. Those analyzed in this study are labeled “*”.
